# How Shell Add-On Products Influence Varsity Football Helmet Performance?

**DOI:** 10.1007/s10439-024-03627-5

**Published:** 2024-10-02

**Authors:** Nicole E.-P. Stark, Mark T. Begonia, Caitlyn Jung, Steven Rowson

**Affiliations:** 1 Department of Biomedical Engineering and Mechanics, Virginia Tech, Blacksburg, USA; 2 Institute for Critical Technology and Applied Science, Virginia Tech, 325 Stanger St., Kelly Hall 120, Blacksburg, VA 24061 USA

**Keywords:** Shell Add-On, Football helmet, Guardian Cap, SAFR Helmet Cover, Concussion

## Abstract

**Purpose:**

The study purpose was to investigate the laboratory-based performance of three commercially available shell add-on products under varsity-level impact conditions.

**Methods:**

Pendulum impact tests were conducted at multiple locations (front, front boss, rear, side) and speeds (3.1, 4.9, 6.4 m/s) using two helmet models. Tests were performed with a single add-on configuration for baseline comparisons and a double add-on configuration to simulate collisions with both players wearing shell add-ons. A linear mixed-effect model was used to evaluate peak linear acceleration (PLA), peak rotational acceleration (PRA), and concussion risk, which was calculated from a bivariate injury risk function, based on shell add-on and test configuration.

**Results:**

All shell add-ons decreased peak head kinematics and injury risk compared to controls, with the Guardian NXT producing the largest reductions (PLA: 7.9%, PRA: 14.1%, Risk: 34.1%) compared to the SAFR Helmet Cover (PLA: 4.5%, PRA: 9.3%, Risk: 24.7%) and Guardian XT (PLA: 3.2%, PRA: 5.0%, Risk: 15.5%). The same trend was observed in the double add-on test configuration. However, the Guardian NXT (PLA: 17.1%; PRA: 11.5%; Risk: 62.8%) and SAFR Helmet Cover (PLA: 12.2%; PRA: 9.1%; Risk: 52.2%) produced larger reductions in peak head kinematics and injury risk than the Guardian XT (PLA: 5.7%, PRA: 2.2%, Risk: 21.8%).

**Conclusion:**

In laboratory-based assessments that simulated varsity-level impact conditions, the Guardian NXT was associated with larger reductions in PLA, PRA, and injury risk compared to the SAFR Helmet Cover and Guardian XT. Although shell add-ons can enhance head protection, helmet model selection should be prioritized.

## Introduction

There is growing concern over the long-term health deficits associated with repetitive head trauma in American football at all levels of competition. This has led to the adoption of various preventative measures designed to promote player safety. Examples include rule changes, revised coaching techniques, and advancements in helmet design [1–7]. The National Operating Committee on Standards for Athletic Equipment (NOCSAE) was formed in the 1970s to certify football helmets by ensuring they could attenuate the impact forces and head accelerations associated with an elevated risk of fatal head injury. In the 2000s, manufacturers began shifting their design efforts to produce helmets that mitigated concussion risk [8]. Researchers have shown that football helmet models that effectively reduced kinematic measures in a laboratory setting were associated with lower rates of concussion on-field [7, 9–13]. Numerous football programs have also begun to use helmet shell add-ons to further alleviate the rising concern for player safety. These products typically consist of an externally mounted layer of padding designed to further dampen the transmission of impact forces into the helmet and, in turn, reduce head acceleration. This technology has rapidly gained popularity for practice in recent years, with certain versions approved for in-game use.

Guardian Sports (Peachtree Corners, GA, USA) and SAFR (Chester Springs, PA, USA) are two companies that developed commercially available helmet shell add-on products. Guardian Sports sells the Guardian Cap, which consists of a soft-shelled, closed-cell foam covered in spandex fabric that is mounted onto the helmet using straps near the facemask and the back. Currently, two versions of the Guardian Cap are available to consumers, with the Guardian Cap XT marketed as the standard version for multiple levels of competition and the Guardian Cap NXT designed for use by players in the National Football League (NFL). SAFR sells the SAFR Helmet Cover, which consists of a polyurethane foam without a fabric overlay mounted onto the helmet using retractable metal hooks at the front and back. The previous version of this product was known as the ProTech Helmet Cap but has since been reformulated and rebranded as the SAFR Helmet Cover. In terms of weight, the SAFR Helmet Cover (1.2 lb) is relatively heavier than the Guardian Cap XT (0.4 lb) and Guardian Cap NXT (0.7 lb).

Although widely adopted, helmet shell add-ons have shown variable performance in the literature [14–17]. In a study by Zuckerman et al., helmets equipped with experimental energy-dissipating pods constructed of varying material properties were tested using a pneumatic ram [14]. They noted that differences in material properties influenced linear and rotational acceleration. When testing commercially available shell add-on products, Breedlove et al. found that when testing Guardian Caps using a standard NOCSAE drop tower, there was no reduction in head acceleration [15]. In a separate study by Cecchi et al., there was a reduction in head acceleration and brain injury risk metrics when the helmets and pneumatic ram’s impactor face were both fitted with Generation 2 Guardian Caps [16]. Likewise, Bailey et al. conducted pneumatic ram testing under impact conditions informed by the NFL. They showed that the Guardian Cap NXT and the ProTech Helmet Cap enhanced helmet performance, with the Guardian Cap NXT outperforming the ProTech Helmet Cap [17]. Bailey et al. also evaluated the influence of helmet shell add-ons applied to both the impactor face and impacted helmets [17]. Their findings indicated that when outfitted with the same add-on, the Guardian Cap NXT and the ProTech Helmet Cap effectively reduced the Head Acceleration Response Metric (HARM) score. However, these studies also demonstrated that helmet shell add-ons’ benefits depended on the helmet model selected [16, 17]. While extensive testing was conducted, it is important to note that these simulated impacts by Cecchi et al. and Bailey et al. were representative of on-field impacts at the NFL level [16, 17] and may not necessarily align with laboratory-based impact performance at the collegiate, high school, and youth levels of competition [11, 12, 18].

These helmet shell add-ons have become popular in practice settings at multiple levels of competition. Prior to the 2023 season, Guardian Caps were mandatory during practices for NFL linemen and linebackers due to their designation as the position groups exposed to the most head contact. At the start of the 2023 season, the NFL expanded the mandate to include running backs and fullbacks and to cover contact practices in the pre-season, regular season, and post-season [19]. The NFL further expanded the mandate to allow all NFL players to wear Guardian Caps during the 2024 season [20]. Meanwhile, the Guardian Cap and SAFR Helmet Cover can be worn in practices and games governed by the National Federation of State High Schools Association (NFHS) and the National Collegiate Athletic Association (NCAA) [21]. However, in a position statement by the National Athletic Trainers’ Association (NATA), Scwartz et al. noted that “helmet add-on products may overstate injury prevention benefits” and recommended that stakeholders exercise caution before investing in them [1].

Although leagues across all levels of play have adopted helmet shell add-on products, the effectiveness of these products is still not fully understood. This study aimed to address the gaps of helmet shell add-on performance at lower levels of competition, such as collegiate and high school. This study had two primary objectives. The first objective was to investigate the performance differences among three commercially available shell add-ons (Guardian Cap XT, Guardian Cap NXT, and SAFR Helmet Cover) using a helmet testing procedure with impact conditions representative of varsity-level competition. The second objective was to evaluate the performance differences associated with double add-on impacts. This study aims to shed light on the overall efficacy of these add-ons in the context of varsity football.

## Methods

**Fig. 1:**
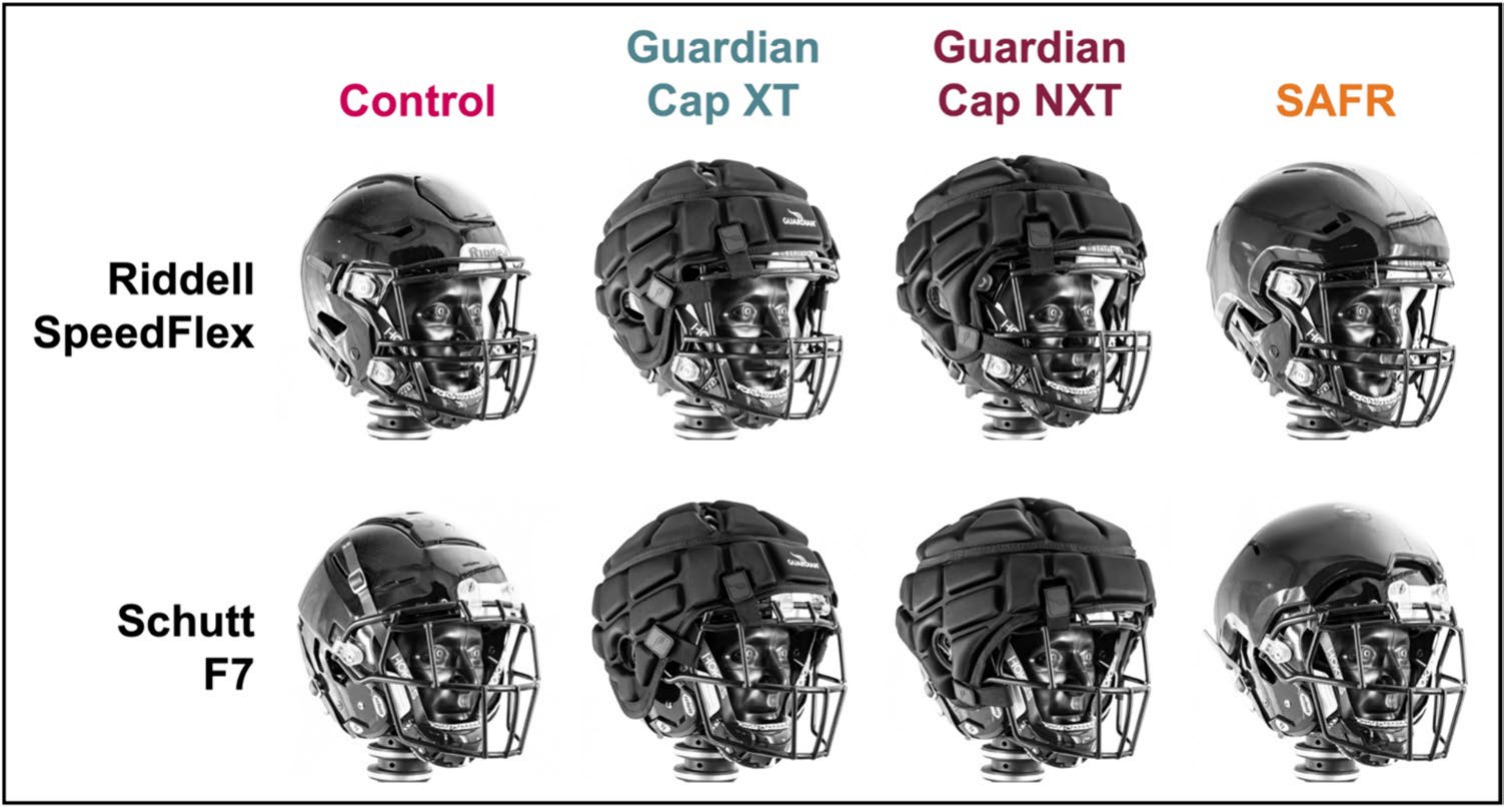
Helmet models with each of the shell add-on products tested.

The Riddell SpeedFlex (Riddell, Des Plaines, IL) and the Schutt F7 VTD (Schutt Sports, Litchfield, IL) were selected for testing in addition to the Guardian Cap XT, Guardian Cap NXT, and the SAFR Helmet Cover. The thickness of the shell add-ons varied based on model and impact location (Table 1). We tested each helmet in a control condition first (i.e., without any shell add-on), followed by a single add-on condition and then a double add-on condition. We followed manufacturer guidelines to ensure each add-on product was secured properly onto each helmet shell (Fig. 1). For the double add-on condition, the Guardian Cap XT, Guardian Cap NXT, and the SAFR Helmet Cover were all mounted securely onto the impactor face. Both versions of the Guardian Cap were cut along the seams so they could be secured tightly over the convex impactor surface. Meanwhile, a section of the SAFR Helmet Cover was carved out and then mounted directly onto the impactor face using adhesive. If necessary, minor adjustments were made to ensure that the add-ons mounted on the helmet and/or the impactor face would produce impacts involving the foam padding.

**Table 1:** Measured shell add-on thickness (mm) based on model and impact location.

Add-on	Front	Front boss	Rear	Side
Guardian XT	16	15	14	15
Guardian NXT	21	21	23	20
SAFR (Riddell)	16	17	13	13
SAFR (Schutt)	13	17	10	13

A pendulum system was used to evaluate helmet performance. A curved nylon impactor face with an 8 in. diameter and 5 in. radius of curvature was attached to the pendulum arm to simulate the curved surface of a football helmet. We conducted tests using the medium NOCSAE headform with a 50th-percentile male Hybrid III neck [22]. The medium NOCSAE headform (Southern Impact Research Center, Rockford, TN) is representative of a 50th-percentile male athlete; its anthropomorphic features enable a more realistic fit between the headform and helmet [23]. The headform and neck assembly were mounted on a sliding mass representing the effective torso mass of a 50th-percentile male. This sliding mass was part of a target table (Biokinetics, Ottawa, Ontario, Canada) that allows linear and rotational head motion to be produced upon impact.

We measured linear and rotational head kinematics using a six-degree-of-freedom sensor package mounted at the NOCSAE headform center of gravity (CG). Relative to headform landmarks, the CG of the medium NOCSAE headform is approximately 1 inch superior to the basic plane and 0.25 inch anterior to the coronal plane. The sensor package consisted of three accelerometers (Endevco 7264B, 2000 g ± 1%, PCB Piezotronics, Depew, NY) and a triaxial angular rate sensor (DTS ARS3 PRO, 18 k deg/s ± 2%, Diversified Technical Systems, Seal Beach, CA). Data were sampled at 20,000 Hz and filtered using a 4-pole Butterworth low pass filter in accordance with SAE J211 specifications. We applied a cut-off frequency of 1650 Hz (CFC 1000) to accelerometer data and a cut-off frequency of 300 Hz (CFC 180) to angular rate sensor data before calculating the resultant peak linear acceleration (PLA), resultant peak rotational acceleration (PRA), and concussion risk. The injury risk function (Eq. 1) was derived from a multivariate logistic regression analysis of data that was collected from football players instrumented with helmet-mounted sensors and then paired with diagnosed concussions [10]. The bivariate risk function considers both PLA ($a$) and PRA ($\alpha$), because linear and rotational components are often considered in assessments of injury risk [12, 13, 24, 25].

**Fig. 2:**
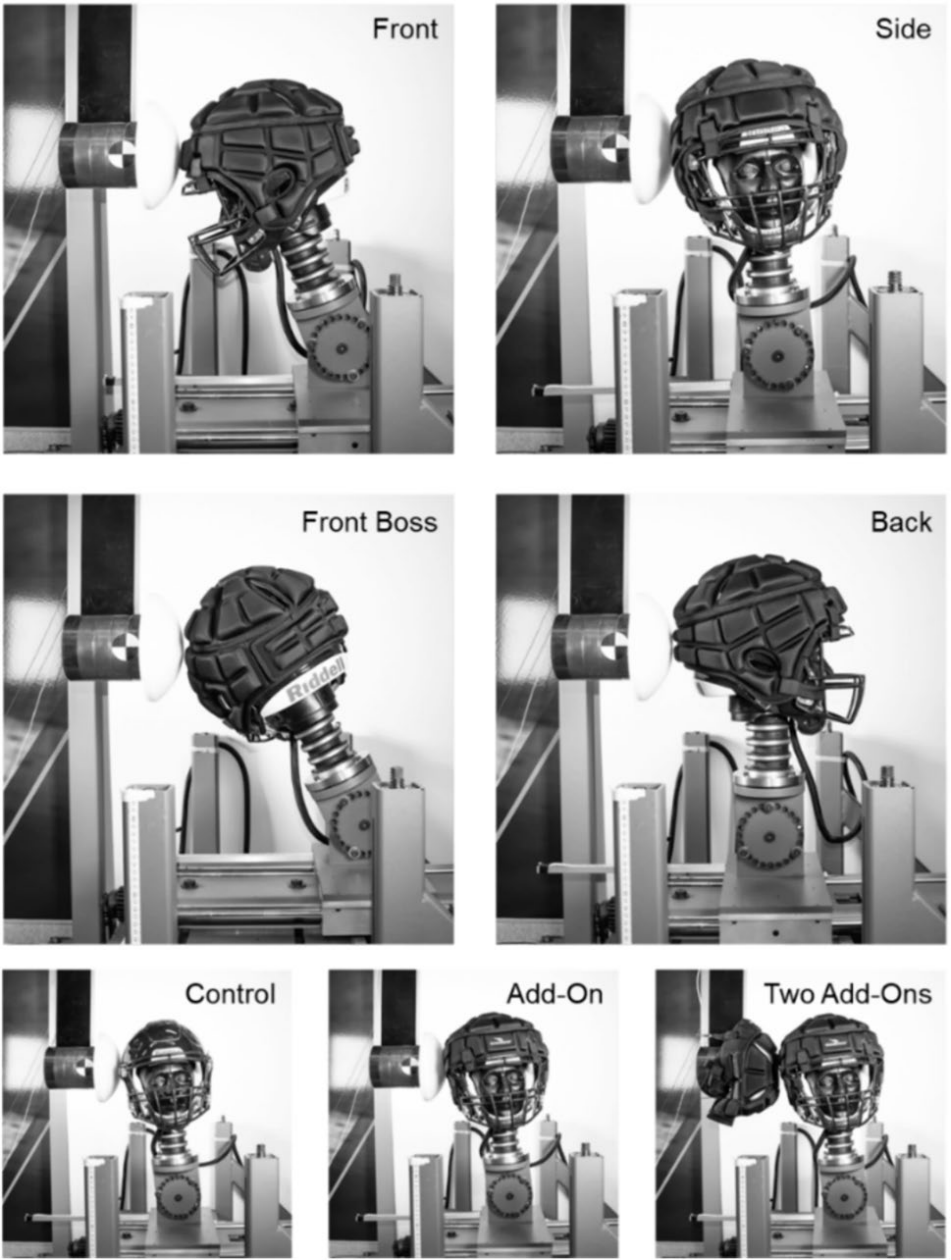
Summary of impact locations and shell add-on test configurations.

The shell add-on testing protocol included four impact locations (front, front boss, side, and back) at impact speeds of 3.1, 4.9, and 6.4 m/s (Fig. 2). Each impact location and impact speed combination were performed twice. This yielded 48 control tests, 144 single add-on tests, and 144 double add-on tests, bringing the total number of tests to 336.

1 $$R\left(a,\alpha \right)=\frac{1}{1+{e}^{-(-10.2+0.0433a+0.000873\alpha -0.00000092a\alpha )}}.$$

We used R (Version 3.3.0, RStudio; Boston, Massachusetts, USA) to evaluate PLA, PRA, and concussion risk using linear mixed-effect regression (LMER) (lmerTest Package [26]), with a significance level *α* < 0.05. Separate LMER models compared location, speed, and helmet shell add-on, including helmet type as a random effect for PLA, PRA, and concussion risk. A second set of LMER models compared location, speed, and helmet shell added to the impactor face, including helmet type as a random effect for PLA, PRA, and concussion risk. Post hoc comparisons were completed using least squares means (lmerTest Package [26]) to compare shell add-on and impactor add-on across conditions.

## Results

### Helmet Shell with Add-On

The addition of helmet shell add-ons led to an average 5.2% reduction in PLA compared to helmets without add-ons (confidence interval [CI] 2.5–7.9%, *p* = 0.142) (Table 2). Helmet shell add-ons also reduced PRA on average by 9.4% (CI 4.4–14.3%, *p* = 0.454). Reductions in peak head kinematics led to an average 24.7% decrease in concussion risk (CI 14.2–35.2%, *p* = 0.716).

**Table 2:** Percent reduction of the mean from control to different helmet shell add-ons

Add-on	Linear accel. (%)	*p*	Rotational accel. (%)	*p*	Risk (%)	*p*
Guardian XT	3.2	0.351	5.0	0.583	15.5	0.614
SAFR	4.5	0.186	9.3	0.307	24.7	0.421
Guardian NXT	7.9	**0.022**	14.1	0.124	34.1	0.269

Bold *p*-values indicate significance

*p* < 0.05

**Fig. 3:**
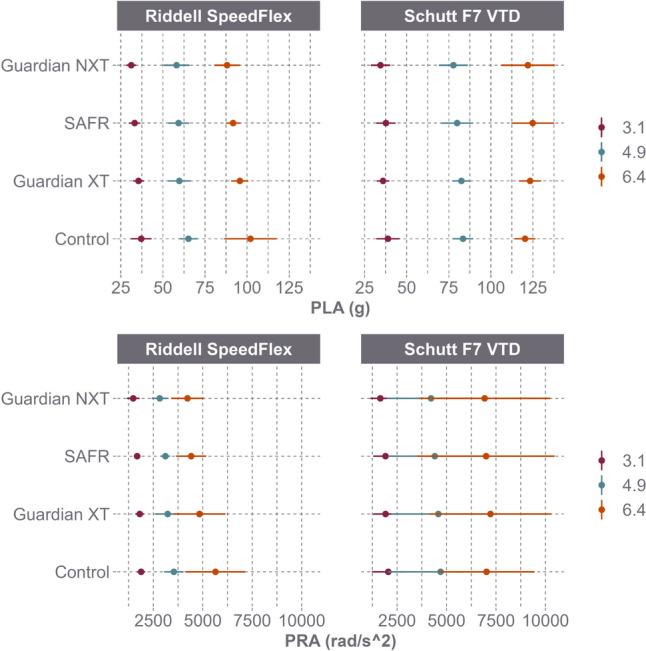
Peak linear acceleration (PLA) and peak rotational acceleration (PRA) (mean and 95% CI) between helmet model and helmet shell add-on across speed

**Fig. 4:**
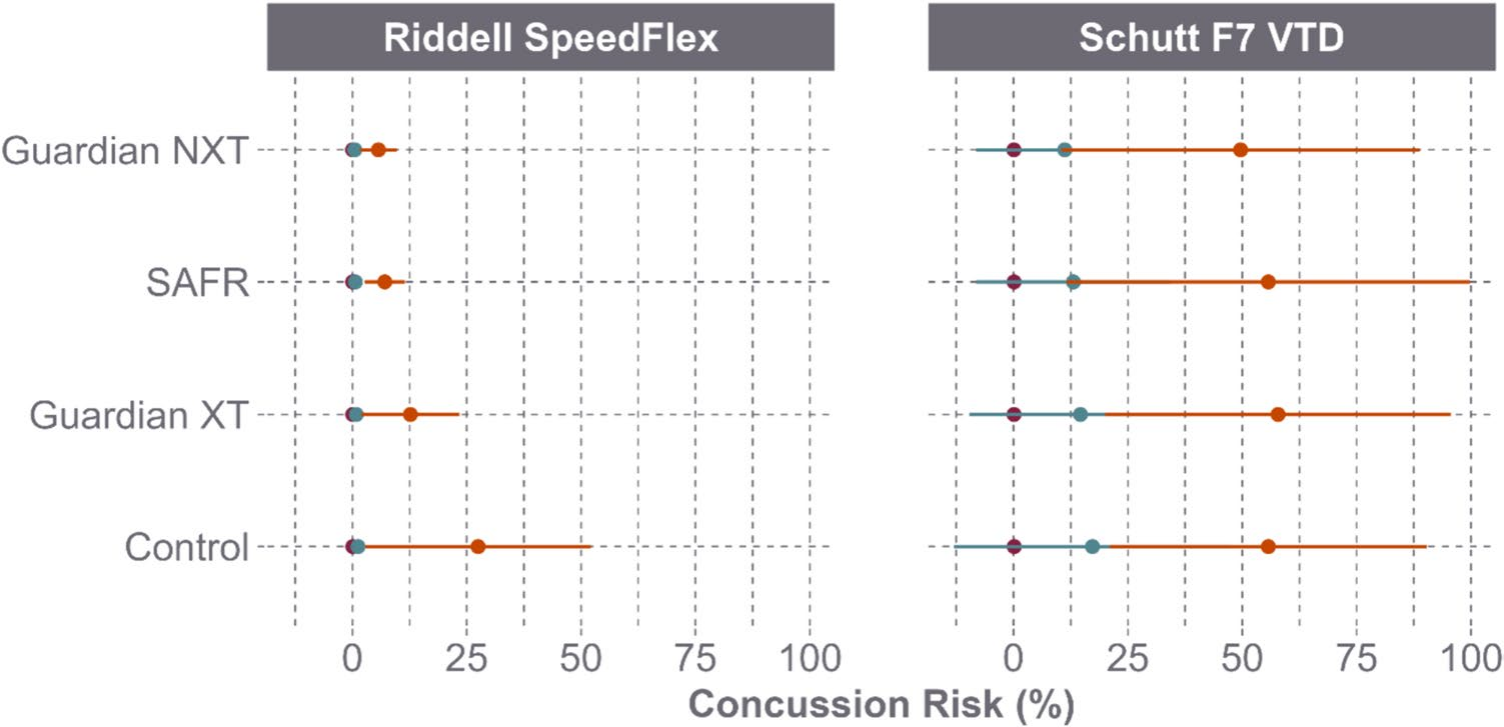
Concussion risk (mean and 95% CI) between helmet model and shell add-on across speed

PLA and PRA response varied by helmet model (Fig. 3). For the Riddell SpeedFlex, the Guardian Cap XT, SAFR Helmet Cover, and Guardian Cap NXT resulted in PLA reductions of 6.6%, 9.8%, and 13.2%, respectively, and PRA reductions of 10.8%, 16.8%, and 22.9%, respectively. As a result of the reductions, the concussion risk for the Riddell SpeedFlex decreased by 52.8% with the Guardian Cap XT, 72.9% with the SAFR Helmet Cover, and 78.3% with the Guardian Cap NXT (Fig. 4). For the Schutt F7 VTD, these add-ons resulted in PLA reductions of 0.4%, 0.1%, and 3.5%, respectively, and PRA reductions of 0.4%, 3.3%, and 7.1%, respectively. As a result of the reductions, the concussion risk for the Schutt F7 VTD decreased by 0.8% with the Guardian Cap XT, 5.8% with the SAFR Helmet Cover, and 16.7% with the Guardian Cap NXT (Fig. 4).

### Impactor and Helmet Shell with Add-on

When both the impactor and the helmets were equipped with helmet shell add-ons, reductions in linear and rotational accelerations were greater for most cases (Table 3). Linear acceleration was reduced by an average of 11.6% (CI 2.4–18.9%, *p* < 0.001), while rotational acceleration was reduced by 7.6% (CI − 1.0 to 28.9%, *p* = 0.009). Reductions in these measures of peak head kinematics led to average decreases in concussion risk by 45.6% (CI 7.4–74.9%, *p* = 0.079).

**Table 3:** Percent reduction of the mean between control tests to both the impactor and the helmets equipped with the same helmet shell add-ons

Add-On	Linear accel. (%)	*p*	Rotational accel. (%)	*p*	Risk (%)	*p*
Guardian XT	5.7	0.086	2.2	0.627	21.8	0.457
SAFR	12.2	** < 0.001**	9.1	**0.031**	52.2	0.078
Guardian NXT	17.1	** < 0.001**	11.5	**0.003**	62.8	**0.033**

Bold *p*-values indicate significance

*p* < 0.05

**Fig. 5:**
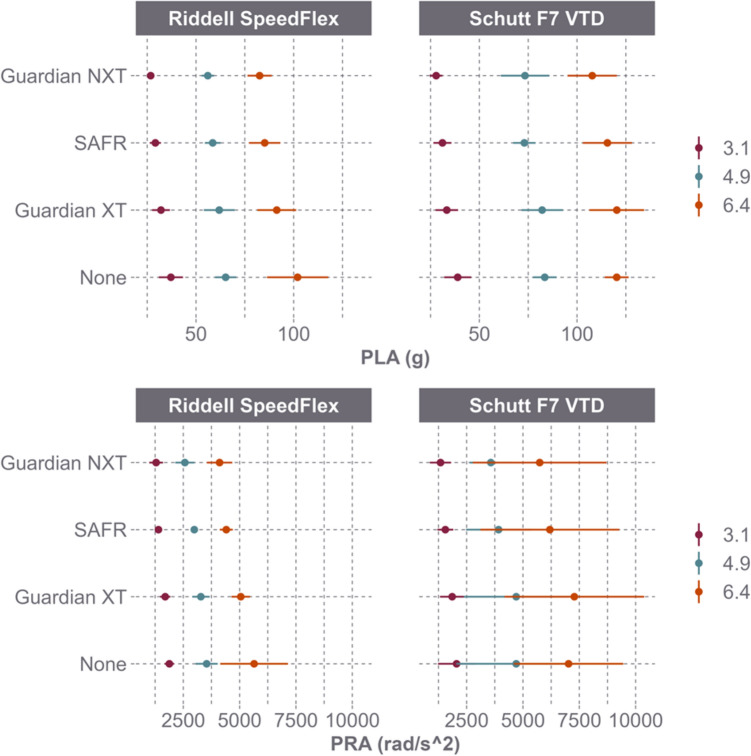
Peak linear acceleration (PLA) and peak rotational acceleration (PRA) (mean and 95% CI) for Riddell SpeedFlex and Schutt F7 VTD across speed when both the impactor and the helmets were equipped with the same shell add-on products

**Fig. 6:**
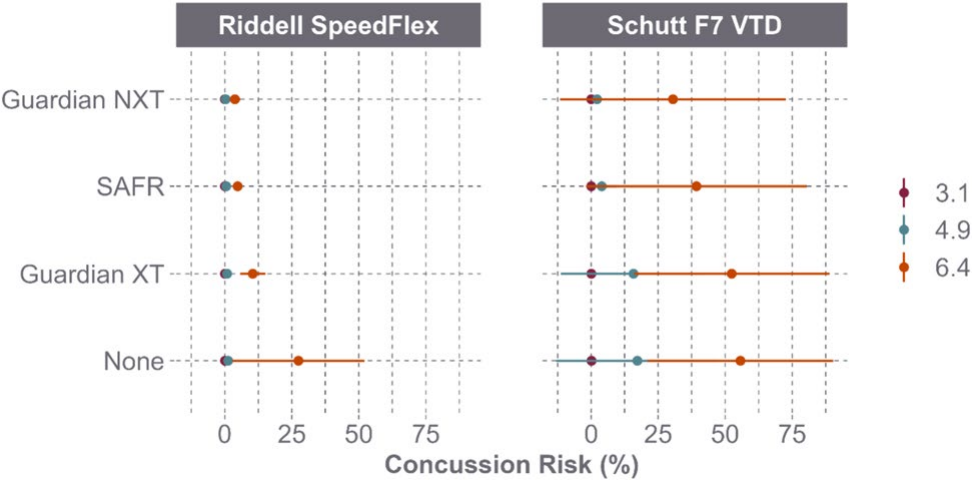
Concussion risk (mean and 95% CI) for Riddell SpeedFlex and Schutt F7 VTD across speed when both the impactor and the helmets were equipped with the same shell add-on products

PLA and PRA reductions were also assessed by helmet model when both the impactor face and the helmet shell were equipped with add-ons (Fig. 5). For the Riddell SpeedFlex, the Guardian Cap XT, SAFR Helmet Cover, and Guardian Cap NXT resulted in average PLA reductions of 9.3%, 15.4%, and 19.1%, respectively, and PRA reductions of 9.4%, 20.5%, and 27.9%, respectively. As a result of the reductions, the concussion risk for the Riddell SpeedFlex decreased by 60.6% with the Guardian Cap XT, 81.3% with the SAFR Helmet Cover, and 85.5% with the Guardian Cap NXT (Fig. 6). For the Schutt F7 VTD, the same three add-ons produced a 2.8%, 9.5%, and 1.5% decrease in PLA, respectively, as well as a 0.4% increase, 15.3% decrease, and 22.6% decrease in PRA, respectively. As a result of the reductions, the concussion risk for the Schutt F7 VTD decreased by 6.6% with the Guardian Cap XT, 40.8% with the SAFR Helmet Cover, and 57.9% with the Guardian Cap NXT (Fig. 6).

## Discussion

The purpose of this study was to evaluate how three commercially available football helmet shell add-on products performed using a helmet testing procedure with impact conditions representative of varsity-level competition. All three shell add-ons reduced headform PLA compared to the control helmet condition. Of the three shell add-on products, the Guardian NXT decreased PLA the most (Table 2). This could be attributed to the NXT version of the Guardian Cap having a 5–9 mm thicker foam padding at different impact locations than the XT version. Breedlove et al. conducted standard NOCSAE drop tests and showed that the Guardian Cap did not significantly reduce PLA or Severity Index (SI) in nearly all impact scenarios despite testing three helmet models at six locations and three impact speeds [15]. They also noted the possibility of the foam padding becoming fully compacted during each test and that only select pieces of padding could be engaged for a given impact. Comparisons of PLA results from Breedlove et al. and Cecchi at al. were also completed using data from trials at 5.47 m/s and 5.5 m/s, respectively, to maintain a consistent impact speed between studies. Additionally, the average PLAs with and without the Guardian Cap were determined by combining results across all helmet models and impact locations at the selected speeds. Both studies showed that average PLAs were lower with Guardian Caps versus controls, with Breedlove et al. reporting a 4.8% reduction and Cecchi et al. reporting a 1.4% reduction [16]. These average percent reductions in PLA are in line with the 3.2% percent reduction we found across all helmets and impact speeds for the Guardian Cap XT (Table 2). A similar comparison could not be made for Bailey et al., because the results were presented using metrics, such as DAMAGE and HARM, which were determined from a combination of PLA and other variables [17]. It is possible that the Guardian XT tested in this study is similar to the version tested by Breedlove et al., given the minimal decrease in PLA observed. However, these results may also highlight the potential effects of test method selection in laboratory-based studies of shell add-on performance. Traditional drop tower tests evaluate helmet performance based primarily on linear acceleration measurements, while test methods involving a pneumatic ram or pendulum enable an assessment of linear and rotational head kinematics as well as corresponding injury metrics that account for both kinematic measures. Consequently, drop tests may only allow for analysis of the impact attenuation of linear forces in a shell add-on. In contrast, pneumatic rams or pendulums enable assessments of other impact mitigation mechanisms, such as the independent movement between the shell add-on product and the helmet outer shell (i.e., decoupling).

All three shell add-on products also reduced headform PRA compared to the control helmet condition, with the Guardian NXT decreasing PRA the most (Table 2). The SAFR Helmet Cover’s polyurethane foam likely possesses different force attenuation properties compared to the padding inside the two Guardian Cap products. Previous work has shown that adding outer shell material to an existing football helmet reduces PRA. Zuckerman et al. completed pneumatic ram testing and showed that energy-dissipating pods constructed with the hardest material (70-duro Sorbothane) reduced rotational acceleration at the front boss location by 49.8% [14]. Differences in material properties between shell add-on covers could partly explain why the SAFR Helmet Cover outperformed the Guardian XT but not the Guardian NXT in the context of reducing rotational head kinematics. The padding in the Guardian NXT is also thicker (Table 1) than the padding in the Guardian XT, which could explain why the Guardian XT reduced PRA the least. Another factor that could be considered is the decoupling between the shell add-on and the helmet. Guardian products can slightly decouple relative to the helmet shell because they are connected via Velcro straps near the facemask and back of the helmet. However, the SAFR Helmet Cover is more rigidly mounted with two retractable metal hooks each at the front and back of the helmet. Cecchi et al. also observed the decoupling between Guardian Caps and helmets in their pneumatic ram tests and suggested that it was the main source of impact mitigation over linear force attenuation [16]. When comparing different shell add-on products, Bailey et al. showed that compared to baseline HARM values, the Guardian NXT lowered HARM by 9% while the ProTech Helmet Cap lowered HARM by only 5% [17]. The HARM equation considers both linear and rotational acceleration in the form of Head Injury Criterion (HIC) and Diffuse Axonal Multi-Axis General Evaluation (DAMAGE), respectively. The bivariate injury risk function used in this study also considers resultant peak linear acceleration (PLA) and peak rotational acceleration (PRA). Compared to control helmet conditions, the Guardian NXT decreased concussion risk the most (34.1%) (Table 2). Reductions in concussion risk were larger relative to the reductions in HARM observed by Bailey et al. because of the risk function’s non-linearity. Both studies showed that the Guardian NXT outperformed other shell add-on products in terms of reducing bivariate injury metrics.

The secondary purpose of this study was to evaluate the performance differences associated with double add-on impacts. The double add-on test configuration was included in this study, because it recreated potential head impacts if the use of shell add-on technology was mandated for all players, not selected positions based on impact exposure. In pendulum impact tests involving two add-on technologies, both the Guardian NXT and SAFR Helmet Cover reduced PLA by over 12% and PRA by over 9% (Table 3). Interestingly, the double add-on test configuration with Guardian XT led to a reduction of less than 5.7% in PLA and 2.2% in PRA. This could be attributed to the Guardian XT already being outperformed by the SAFR Helmet Cover and Guardian NXT in terms of reducing PLA and PRA in the single add-on test configuration or the Guardian XT padding experiencing permanent deformation during testing. (Table 2). However, visual inspection of Guardian XT samples when switching between test configurations did not reveal any damage to the underlying padding during testing. A separate series of tests were not conducted to assess the potential decline in shell add-on performance from successive impacts. However, Cecchi et al. performed 100 consecutive pneumatic ram tests with both the impactor face and helmet shell fitted with a shell add-on, and they determined that there was no correlation between the number of impacts and the HARM values calculated [16]. This could be an objective for future studies aimed at determining the efficacy of shell add-on technology.

Facemask impacts were not included in the present study as the shell add-ons are unlikely to affect facemask-to-facemask impacts because the loading path does not engage the padding of the shell add-ons. However, in facemask-to-shell impacts there might be some effect as the loading path would engage the padding. Previous research has noted that conducting tests directly to the facemask resulted in an unrealistic interaction between the facemask and the impactor face as the facemask would bend, which is not typical of facemask impacts in the real world [27]. Additionally, on-field studies have not found differences between facemask and frontal hits due to on-field variance [28].

The Summation of Tests for the Analysis of Risk (STAR) methodology was used as a basis for evaluating the impact performance of shell add-on products [29]. The STAR score is intended to aid consumer purchasing decisions on helmets by consolidating various in-laboratory tests into a single value. The STAR score is the sum of the weighted injury risk values determined from each impact scenario; thus, lower STAR scores indicate better helmet performance [29]. When considering both the Riddell SpeedFlex and Schutt F7 VTD in the single add-on configuration, we found that the STAR score had an average reduction of 49.7% for the Guardian Cap NXT, 38.1% for the SAFR Helmet Cover, and 27.7% for the Guardian Cap XT. In the double add-on configuration, we found an average reduction in STAR score of 73.8% for the Guardian Cap NXT, 62.1% for the SAFR Helmet Cover, and 25.5% for the Guardian Cap XT. Although these scores were derived from the in-laboratory testing, it is important to note that they also represent a cumulative performance over hundreds of head impacts in a season.

Although different shell add-on products reduced head accelerations, injury risk, and the STAR score, the Riddell SpeedFlex outperformed the Schutt F7 VTD in the control condition (Figs. 3 and 4). The helmet-to-helmet difference remained the dominating factor over the difference between helmets with an add-on and helmets without an add-on. As seen in Figs. 4 and 6, mounting a Guardian Cap NXT onto the Schutt F7 VTD still resulted in a higher average concussion risk than the Riddell SpeedFlex in the control condition. When comparing the corresponding STAR scores, the Schutt F7 VTD with the Guardian Cap NXT had a STAR score of 9.31 in the single add-on condition and 4.19 in the double add-on condition, whereas the Riddell SpeedFlex with no shell add-on had a score of 4.15. This suggests that mounting a shell add-on product on a poor-performing helmet model will not create a better performing helmet and that top performing helmets without add-ons would still offer better risk reduction compared to poor-performing helmets with add-ons.

This study had limitations that should be noted. First, there may be some real-world impact scenarios that were not covered by the laboratory helmet testing protocol used in this study. However, the protocol was considered to be robust because it encompassed a broad range of head impact conditions that were derived from on-field data collected from players wearing football helmets instrumented with sensors. Second, we did not conduct a series of tests to assess the potential degradation of shell add-on performance, although Cecchi et al. determined from consecutive impact tests that there was no correlation between the impact count and an injury metric that considers both linear and rotational head kinematics [16]. Third, only two football helmet models were chosen based on the authors’ assessment of their popularity at multiple levels of competition. Studies by Breedlove et al., Bailey et al., and Cecchi et al. were all conducted using helmet models from at least two different helmet manufacturers [15–17]. Given our emphasis on examining the performance of three shell add-on products, we focused on only two helmets from two separate companies rather than a larger selection with a single representative model from all manufacturers. Fourth, durability and useful life of the shell add-on products through various factors such as temperature, relative humidity, and time was not examined in the present study and should be explored in future. Finally, only the same brand and model of shell add-on were impacted in the double add-on test configuration. Future work could explore the impact responses associated with the interaction of different types of shell add-on products, which could simulate potential impacts if players were allowed to wear any commercially available shell add-on approved by league officials.

This study investigated the performance of three commercially available shell add-on products (Guardian Cap XT, Guardian Cap NXT, and SAFR Helmet Cover) with a helmet testing protocol that replicated on-field impact conditions at the varsity level of competition. The present study provides novel information on how the implementation of shell add-on products influences the peak head kinematics and injury risk within football at a varsity-level competition. These products were first evaluated under a single add-on test configuration to compare peak head kinematics and injury risk versus baseline results from two different helmet models. These products were then evaluated under a double add-on test configuration to simulate league-mandated use in which both players in a head collision would be wearing a shell add-on product. All three shell add-ons reduced peak head kinematics and injury risk compared to the control helmet condition, with the Guardian NXT outperforming the other two shell add-on products. The same trend was observed in the double add-on test configuration. However, the Guardian NXT and SAFR Helmet Cover produced notably larger reductions in peak head kinematics and injury risk compared to those made by the Guardian XT. This study was a comprehensive laboratory-based assessment of multiple shell add-on products that are currently available to consumers, but it is important to note that the reductions in accelerations are specific to the impact conditions used in our test protocol. Not everyone experiences these same impacts, so individual risk will vary from the averages presented here. Regardless of the helmet or add-on used, any player can still sustain a concussion. However, these results represent average risks across the football player population for a given head impact exposure and should not be interpreted as absolute risk measurements for individual players. Moreover, the effectiveness of these add-ons can vary significantly depending on the helmet model selected because each model possesses different proprietary technologies with varying impact mitigation properties. The helmet model itself is crucial as it sets the baseline level of protection. Although shell add-on products have the capacity to enhance head protection, emphasis should be placed on helmet model selection first due to the range in impact performance of helmet models available to players.
